# Triglycerides as Determinants of Global Lipoprotein Derangement: Implications for Cardiovascular Prevention

**DOI:** 10.3390/ijms26178284

**Published:** 2025-08-26

**Authors:** Núria Amigó, Pol Torné, Liv T. Nordestgaard, Francesco Di Giacomo-Barbagallo, Carla Merino, Paolo Magni, Ana González-Lleó, Natalia Andreychuk, Alberico L. Catapano, Lluís Masana, Daiana Ibarretxe

**Affiliations:** 1 Basic Medical Sciences, Faculty of medicine, Universitat Rovira i Virgili, 43003 Tarragona, Spain; namigo@biosferteslab.com; 2 Institut Investigació Sanitaria Pere Virgili, 43204 Reus, Spain; 3 Biosfer Teslab, 43204 Reus, Spain; ptorne@biosferteslab.com (P.T.); cmerino@biosferteslab.com (C.M.); 4 Department of Clinical Biochemistry, Copenhagen University Hospital–Herlev and Gentofte, BorgmesterIb Juuls Vej 1, 2730 Herlev, Denmark; liv.tybjaerg.nordestgaard@regionh.dk; 5 Medical Research Council Integrative Epidemiology Unit, Population Health Sciences, University of Bristol, Bristol BS8 2BN, UK; 6 Unitat de Medicina Vascular i Metabolisme, Hospital Universitari Sant Joan, 43204 Reus, Spain; fdigiacomobarbagallo@gmail.com (F.D.G.-B.); agonzalezlleo@gmail.com (A.G.-L.); 7 Department of Clinical and Experimental Medicine, Internal Medicine Garibaldi-Nesima Hospital, University of Catania, 95122 Catania, Italy; 8 Department of Pharmacological and Biomolecular Sciences, Università degli Studi di Milano, 20133 Milan, Italy; paolo.magni@unimi.it (P.M.); alberico.catapano@unimi.it (A.L.C.); 9 Multimedica IRCCS, Sesto S. Giovanni, 20099 Milan, Italy; 10 Unitat de Recerca en Lipids i Arteriosclerosis, Universitat Rovira i Virgili, 43201 Reus, Spain; natalia.andreychuk@salutsantjoan.cat (N.A.); daiana.ibarretxe@urv.cat (D.I.); 11 Centro de Investigación Biomédica en Red Diabetes y Enfermedades Metabòlicas (CIBERDEM), Instituto de Investigación Carlos III, 28029 Madrid, Spain

**Keywords:** triglycerides, cardiovascular prevention, nuclear magnetic resonance, advanced lipoprotein profile, residual cardiovascular risk, cholesterol

## Abstract

Plasma triglyceride levels are a strong cardiovascular risk marker. However, triglyceride–lowering therapies have not demonstrated a reduction in cardiovascular events, suggesting that triglycerides may serve as surrogate markers for other atherogenic mechanisms. The aim is to investigate the role of triglycerides in derangements of the global lipoprotein profile, as assessed by ^1^H–NMR spectroscopy. Serum lipoprotein profiles were analyzed in patients with metabolic alterations attending the Lipid Unit of a University hospital (*n* = 822). Lipoprotein particle number, size, and composition were evaluated and visualized through a graphic representation of a lipoprotein network analysis referred to lipid silhouette in patients sorted by triglyceride quartiles. Profound alterations in lipoprotein quantity and composition were associated with incremental triglyceride concentrations independently on the presence of diabetes or obesity, including a significantly increased number of VLDL and smaller LDL particles, and higher remnant cholesterol, representing up to 30% of all cholesterol in quartile 4. It was also a significant triglyceride enrichment of LDL and HDL particles. Triglycerides are not merely components of atherogenic dyslipidemia; they are a key driver of the overall changes and are strongly associated with a proatherogenic plasma lipoprotein profile. Visualization of these alterations provides supplementary insights to support cardiovascular prevention strategies.

## 1. Introduction

Low-density lipoproteins (LDL) play a causal role in atherosclerotic cardiovascular disease (ASCVD), making LDL reduction a cornerstone in the prevention and treatment of atherosclerosis [1]. However, ASCVD events still occur in individuals with very low levels of LDL cholesterol (LDL–C). In the Fourier OLE study, over 2000 patients attained an LDL–C concentration below 0.5 mmol/L (20 mg/dL), yet up to 10% experienced cardiovascular events [2]. This persistent cardiovascular risk beyond LDL–C can be attributed to various factors. Some are independent of lipoproteins, including inflammation, oxidation and prothrombotic state [3]. On the other hand, because atherosclerosis is a consequence of a cumulative exposure of LDL–C, there is a baseline risk difficult to modify at later stages [4]. However, an LDL-independent cardiovascular risk associated with lipoproteins, arising from alterations in particle metabolism, should be considered. Among them, the role of lipoprotein(a) [Lp(a)] has emerged as a strong CV risk marker. Its concentration is in large part determined genetically; thus, their plasma levels are a key consideration for certain patients [5]. A high triglyceride (TG) and low HDL cholesterol pattern, commonly referred to as atherogenic dyslipidemia, is a frequent lipoprotein alteration associated with increased cardiovascular risk [6]. This clinical phenotype reflects a deeper derangement in lipoprotein metabolism, encompassing both quantitative and qualitative abnormalities [7]. TG-rich lipoprotein (TRL) particles, with diameters up to 70 nm—including very low-density lipoproteins (VLDL), TRL remnants, and intermediate-density lipoproteins (IDL)—are atherogenic and capable of crossing the endothelial layer of arteries [8]. This lipoprotein-associated risk, beyond LDL–C, can be detected indirectly through markers such as non-HDL cholesterol, apolipoprotein B (apoB), or remnant cholesterol. Numerous studies have demonstrated the utility of these parameters in defining cardiovascular risk [9,10]. Excess apoB relative to LDL concentrations has also been linked to increased cardiovascular risk, underscoring the direct contribution of TRL particles [11].

Paradoxically, therapies targeting TG reduction and HDL–C elevation—such as fibrates, niacin, and cholesteryl ester transfer protein (CETP) inhibitors—have failed to deliver consistent clinical benefits [12,13,14]. Recent findings from the PROMINENT study revealed that despite significant reductions in TRL and their components through pemafibrate treatment, the overall impact on ASCVD was negligible. The lack of efficacy has been attributed to the null effect on total apoB-containing particles due to an increase in LDL levels [15]. Omega–3 fatty acids have been proposed as a potential therapeutic approach for reducing cardiovascular risk in hypertriglyceridemia. However, only high doses of highly purified icosapent ethyl have demonstrated clear cardiovascular benefits, predominantly through TG-independent mechanisms [16].

Over the years, our group has analyzed serum lipoproteins using nuclear magnetic resonance (NMR) spectroscopy (Liposcale^®^ test, IVD–CE) in patients attending the lipid clinic for metabolic abnormalities [17]. We have observed that quantitative and qualitative modifications in all lipoprotein subclasses are common, extending beyond standard clinical lipid profiles, including apoB measurements. In clinical practice, elevated serum TG levels are typically associated with global alterations in lipoprotein parameters assessed by NMR.

Our hypothesis is that triglycerides influence widespread derangements in lipoprotein metabolism detected by the presence of alterations in all components of lipoprotein profile and therefore serve as biomarkers of a pro-atherogenic lipoprotein disorder beyond their own concentrations, which can be captured by plasma NMR.

Because the overwhelming and complex data provided by NMR remains a barrier to its routine clinical use, we developed a visual representation, termed the “lipid silhouette” (LS), to depict global lipoprotein alterations identified through serum NMR. The LS comprises a circular visualization incorporating ten NMR lipoprotein parameters [18].

In this study, we aimed to demonstrate the impact of incremental TG concentrations on the global quantitative and qualitative characteristics of circulating lipoprotein particles in a clinical setting by using this novel visualization approach.

## 2. Results

### 2.1. Demographic, Clinical, and Biochemical Characteristics

Table 1 summarizes the main demographic, clinical, and standard biochemical data of patients, stratified by TG quartiles. The mean age of the cohort was 48 years, with 44.5% being female. Obesity and type 2 diabetes mellitus were more prevalent in the highest TG quartile. The median (IQR) of standard lipid values for the whole group were total cholesterol 6.18 (5.17–7.06) mmol/L; LDL–C: 3.92 (2.93–4.68) mmol/L, with minimal variation across TG quartiles; HDL–C: 1.27 (1.03–1.58) mmol/L showing a decline across TG quartiles. The overall TG values were moderately elevated: 1.57 (1.04–2.44) mmol/L achieving 3.79 (2.81–5.48) mmol/L in the fourth quartile.

### 2.2. ^1^H–NMR Lipoprotein Data

The ^1^H–NMR lipoprotein profile data, shown in Table 2, reveal significant alterations across TG quartiles. The total number of VLDL particles increased up to six-fold from quartile 1 to quartile 4, [from 26 (22–32) nmol/L to 160 (124–234) nmol/L] mainly due to TRL remnants (smaller VLDL particles).

While the total number of LDL particles increased modestly [Q1: 1470 (1180–1690); Q4: 1720 (1450–2100)] nmol/L] (approximately 17%), the smaller LDL particles rose a 50% [721 (630–807) nmol/L to 1100 (889–1280) nmol/L], from quartile one to quartile four.

**Figure 1:**
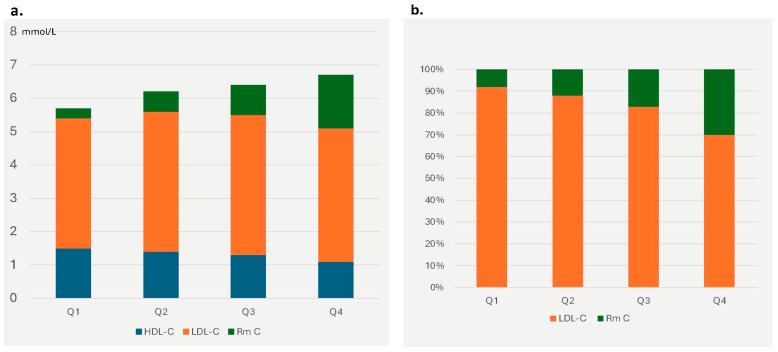
(**a**) Absolute values of total cholesterol concentrations and its distribution between triglyceride-rich lipoproteins (remnant cholesterol—Rm C), low-density lipoproteins (LDL), and high-density lipoproteins (HDL) sorted by quartiles of triglyceride concentrations based on NMR analysis. (**b**) Remnant cholesterol and LDL–C distribution. Percentage distribution of cholesterol across all atherogenic, apolipoprotein B containing, lipoproteins.

The total number of HDL particles decreased with increasing TG levels. TRL carried an increased cholesterol load (remnant cholesterol), which rose more than fourfold across quartiles. In the highest TG quartile, remnant cholesterol constituted approximately 24% of total cholesterol (Figure 1a) and 30% of the cholesterol transported by atherogenic particles, compared to only 8% in the lowest quartile (Figure 1b). Because the number of cholesterol molecules per particle was almost unchanged, this increase can be attributed to the higher number of particles.

Both LDL and HDL particles were enriched in triglycerides at higher TG concentrations. While the cholesterol content in HDL particles showed a decline across TG quartiles, the triglyceride-to-cholesterol ratio (HDL–TG/HDL–Ch) in HDL particles showed a marked increase [Q1: 0.179 (0.141–0.222); Q4: 0.415 (0.285–0.587)].

The mean particle size of VLDL remained constant across TG quartiles. However, both LDL and HDL particle sizes were significantly reduced with increasing TG levels.

**Figure 2:**
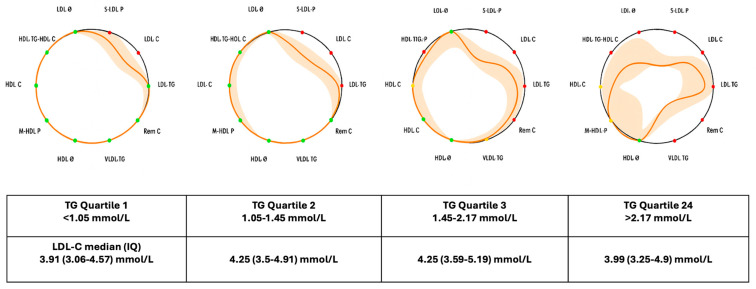
Mean lipid silhouettes derived from ten lipoprotein parameters measured by nuclear magnetic resonance (^1^H–NMR) in patients categorized by triglyceride concentration quartiles. Alteration of parameters are represented as a contraction of the silhouette towards its center, with the extent of contraction proportional to the variance of the respective parameter. LDL–Z: LDL diameter size; S–LDL–P: smaller LDL particles; LDL–C: LDL cholesterol; LDL–TG: LDL triglycerides; Rem C: Remnant cholesterol; VLDL–TG: VLDL triglycerides; HDL–Z: HDL diameter size; M–HDL–P: number of medium-size HDL particles; HDL–C: HDL cholesterol; HDL–TG/HDL–C: HDL triglycerides/cholesterol ratio. The shadow represents the confidence interval.

The ^1^H–NMR data indicated a global proatherogenic lipoprotein profile associated with increasing TG concentrations. These alterations were visually apparent using the lipid silhouette, as shown in Figure 2. The LS displayed progressive circumference contraction and a greater number of affected parameters with higher TG concentrations. In contrast, patients with normal TG levels but elevated LDL–C showed limited variation in ^1^H–NMR profiles, confined to LDL–C and particle number (Supplementary Figure S1).

A similar pattern was observed when patients were stratified based on the presence or absence of both diabetes and obesity (Table 3 and Table 4). As expected, triglyceride concentrations were higher in the D/O group. However, in both subgroups (with and without D/O), the overall alterations in lipoprotein parameters assessed by ^1^H–NMR increased progressively with rising triglyceride levels, mirroring the pattern observed in the total sample (Table 4). Moreover, both women and men exhibit similar alterations in lipoprotein patterns associated with increasing triglyceride concentrations (Supplementary Table S1).

## 3. Discussion

This study demonstrates the profound changes in overall lipoprotein metabolism that are associated with incremental serum TG concentrations. As TG levels increase, there is a significant rise in the number of atherogenic lipoprotein particles, mainly TRL remnants and smaller LDL. In patients within the highest TG quartile (median ^1^H–NMR –TG concentration: 3.25 mmol/L; IQR: 2.53–4.50 mmol/L), TRLs carried 30% of the cholesterol contained in atherogenic particles, compared to 8% in the lowest quartile (Figure 1b) not due to a TG enrichment per particle but because of a higher particle number. Recent evidence indicates that the cardiovascular risk associated with TRL remnants is primarily driven by smaller particles due to their enhanced penetration into the arterial intima. Consistent with these findings, there was a greater increase in the proportion of smaller VLDL particles. This shift may contribute to the heightened atherogenic potential associated with elevated triglyceride levels [19].

According to our plasma ^1^H–NMR study, as expected, LDL and HDL particles undergo substantial changes with elevated TG levels. These particles become enriched in triglycerides and exhibit smaller sizes. Small LDL particles have been shown to possess higher atherogenic potential [20]. Similarly, smaller HDL particles are rapidly removed from circulation, resulting in reduced HDL particle numbers, which could be linked to increased atherogenicity. Additionally, the HDL–TG/HDL–Ch ratio increases significantly with higher TG levels. TG-enriched HDL, as opposed to cholesterol-rich HDL, has been associated with elevated cardiovascular (CV) risk [21,22], probably due, in part, to compromised functionality and structure driven by triglycerides overload, which would be aligned with a previously reported disruption in the molecular order of lipids in triglyceride-enriched HDL [23].

Obesity, diabetes, and insulin-resistant states are generally associated with hypertriglyceridemia. According to our data, although patients with D/O exhibited higher triglyceride levels and more pronounced global lipoprotein alterations, triglycerides were associated with widespread disruptions in lipoprotein metabolism in both groups (with and without D/O) in a dose-dependent manner.

Similar alterations in lipoprotein patterns were also observed in both women and men (Supplementary Table S1).

The replication of global lipoprotein alterations associated with increasing triglyceride concentrations—observed in the total population as well as in men and women, and in individuals with or without diabetes or obesity—reinforces the central role of triglycerides in these alterations.

Because circulating TG levels correlate with intrahepatic TG content, except in conditions characterized by a compromised export of TRL [24], it can be speculated that the mechanism underlying these alterations involves an increased intrahepatic triglyceride availability to apoB, promoting the enhanced synthesis of nascent VLDL particles, which in turn determines the total number of circulating apoB-containing lipoproteins [25]. Moreover, the catabolism of VLDL in the bloodstream is modulated by lipases, primarily lipoprotein lipase (LPL). Under optimal conditions (TG < 1.13 mmol/L), LPL hydrolyzes up to 25 VLDL pools per day, extracting over 80% of fatty acids (FAs) per particle [26]. However, in hypertriglyceridemia, LPL’s activity is reduced to fewer than five pools per day, resulting in partially delipidated TRL with longer half-life and greater atherogenic potential due to defective hydrolysis perhaps associated to an apoE/apoCIII imbalance [27].

These alterations can be captured by plasma ^1^H–NMR analyses and visualized by the LS. This tool is designed to translate the data obtained from NMR lipoprotein analysis into clinically relevant information by reducing the number of parameters and providing an at-a-glance overview. It gives to the clinician a full picture of lipid metabolism modifications in their patients contributing to the clinical decision process. Even small increases in triglycerides can trigger substantial changes across the entire lipoprotein cascade.

Advanced lipoprotein profiling using ^1^H–NMR provides a more detailed view of lipoprotein alterations compared to standard parameters. For example, while apolipoprotein B (apoB) levels were higher in the third and fourth TG quartiles, the increase was less pronounced than the actual rise in the number of atherogenic particles. On the other hand, apo B concentrations give no idea of the type of particle that accumulates in plasma. Notably, these ^1^H–NMR-detected changes emerged even at moderate TG elevations. While alterations were evident starting from the second TG quartile, the highest quartile threshold was just 2.17 mmol/L, with a median TG level of 3.25 mmol/L.

Interesting, these changes are not observed in patients with normal TG levels but elevated LDL–C—such as those with familial or polygenic hypercholesterolemia—that have a limited variation in ^1^H–NMR profiles, confined to LDL–C and particle number (supplementary Figure S1), supporting the differential impact of triglycerides on overall lipoprotein metabolism.

**Figure 3:**
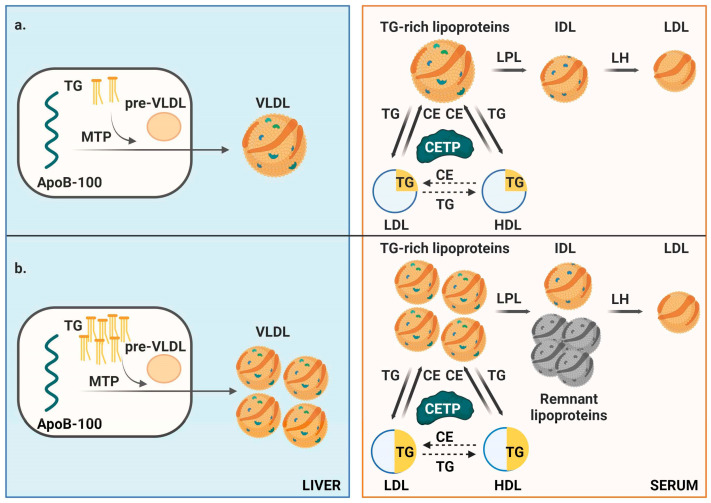
Schematic representation of triglyceride metabolism. (**a**) In individuals with low triglyceride (TG) levels, hepatic production of very low-density lipoprotein (VLDL) particles via microsomal triglyceride transfer protein (MTP) is lower. Once in circulation, triglyceride-rich lipoproteins (TRLs) undergo efficient delipidation, facilitating normal lipid exchange with low-density lipoproteins (LDL) and high-density lipoproteins (HDL) mediated by cholesteryl ester transfer protein (CETP). (**b**) In individuals with high intrahepatic triglyceride content (e.g., due to obesity, diabetes, unhealthy diet, or alcohol consumption) hepatic secretion of VLDL increases, enlarging the pool of atherogenic lipoproteins. The excessive TRL burden overwhelms lipoprotein lipase (LPL) and hepatic lipase (HL) activity, leading to partially delipidated TRLs (remnant lipoproteins). Elevated circulating triglyceride levels disrupt lipid exchange processes mediated by cholesteryl ester transfer protein (CETP), leading to triglyceride enrichment and cholesterol depletion in LDL and HDL particles. This results in the formation of smaller, denser LDL and HDL particles. Concurrently, TRLs transport more cholesterol, increasing levels of remnant cholesterol, which could significantly contribute to atherogenesis.

^1^H–NMR data and the lipid silhouette (LS) clearly illustrate these TG-associated alterations, including increased circulating TRL particles, defective delipidating, remnant cholesterol accumulation, and the formation of small, TG-enriched LDL and HDL particles (Figure 3) [26].

Optimal therapeutic strategies may focus on reducing TG and fatty acid accumulation in the liver and enterocytes, thereby limiting VLDL formation. Liver fat predominantly derives from adipose tissue lipolysis (60%), de novo lipogenesis (25%), and lipoprotein uptake (15%) [28]. Enhancing insulin sensitivity, reducing lipolysis, and promoting FA oxidation or reducing hepatic lipogenesis could prove effective. Lifestyle interventions like physical activity, weight loss, and waist circumference reduction remain the cornerstone recommendations. Diet interventions leading to lower TG concentrations have been associated with advanced lipoprotein profile improvements [29,30]. Promisingly, GLP–1 receptor agonists have shown substantial efficacy in reducing fat mass [31]. High dose Eicosapentaenoic acid has been shown to reduce the number of CV events in hipertriglyceridemic patients, probably by mechanisms independent of TG primarily inflammation, which exerts a direct effect on ASCVD pathogenesis. Although these effects seem to be independent of TG reduction, an impact on other components of lipoprotein metabolism alterations could be taken into consideration [32]. Inflammation, together with elevated concentrations of atherogenic lipoproteins, represents a major pathway in ASCVD pathogenesis, and both processes may be driven by excess intrahepatic triglyceride accumulation.

This study has several limitations. The sample size and patient characteristics may not be representative of all metabolic patients. The LS, while clinically valuable, involves subjective interpretation based on observations and literature data. Additionally, the Liposcale test, though clinically adjusted, is relatively costly and best applied selectively.

These findings are not generalizable to severe genetic hypertriglyceridemia, such as Familial Chylomicronemia Syndrome, which results from defects in lipolysis rather than increased TRL synthesis. Moreover, chylomicron accumulation is poorly detected by ^1^H–NMR.

Although follow-up data for this cohort are lacking, limiting causal inference, a randomized controlled trial of Saroglitazar, a dual PPAR–a/g agonist aimed at reducing hepatic fat in steatotic liver disease, demonstrated that LS detected a global improvement in NMR lipoprotein profile alongside triglyceride reduction, supporting the possible causal link to the observed lipoprotein abnormalities [18].

In brief, hypertriglyceridemia is a strong biomarker of highly atherogenic plasma and probably plays a role in determining global lipoprotein changes. Alterations across all lipoprotein families are comprehensively detected by serum ^1^H–NMR, with the lipid profile serving as a valuable clinical tool for visualizing disruptions in lipid metabolism and guiding therapeutic interventions aimed at more effective cardiovascular prevention.

## 4. Materials and Methods

### 4.1. Patients

This study included 822 adult patients (≥18 years old) of both sexes who attended the lipid unit at our university hospital for evaluation of lipid abnormalities, cardiovascular risk management and metabolic alteration as obesity, metabolic syndrome or diabetes and underwent advanced ^1^H–NMR lipoprotein testing. Data on medical history, anthropometric measurements, and physical examination were collected. Blood samples were obtained after a minimum 12 h fasting period for standard hematological and biochemical analyses. Carotid ultrasonography was performed to measure intima-media thickness and detect the presence of plaques.

### 4.2. ^1^H–NMR Lipoprotein Profiling

Advanced ^1^H–NMR lipoprotein testing was routinely conducted in our lipid unit during the baseline assessment of patients either naïve to lipid-lowering therapy (LLT) or after a 6-week LLT washout period. This testing was indicated for primary prevention patients with severe dyslipidemia (e.g., hypercholesterolemia, mixed dyslipidemia, or hypertriglyceridemia) when the standard lipid profile was insufficient, in accordance with the Spanish consensus on the clinical use of Liposcale^®^ [33]. Secondary prevention patients typically remained on LLT, and washout was unethical. However, a small group of secondary prevention patients not on LLT at the baseline visit was also included.

For ^1^H–NMR lipoprotein analysis, a 200 μL plasma aliquot was analyzed by using NMR spectroscopy. Briefly, we employed a double stimulated echo pulse sequence incorporating bipolar gradient pulses and a longitudinal eddy current delay (LED; ledbpgp2s1d: LED-bipolar gradient pulse sequence). Additionally, one-dimensional Nuclear Overhauser Effect Spectroscopy (1D NOESY) was performed. All sequences were executed under quantitative conditions at 37  °C.

To assess the size and concentration of nine lipoprotein subclasses—namely, large, medium, and small particles of very low-density lipoprotein (VLDL), low-density lipoprotein (LDL), and high-density lipoprotein (HDL)—we utilized the Liposcale^®^ method, which is IVD–CE certified. Cholesterol and TG concentrations in lipoprotein subclasses were also measured. Remnant cholesterol (Rm–Chol) was calculated as the sum of IDL and VLDL cholesterol.

Particle concentrations were estimated based on the amplitude and decay of spectrally resolved methyl group signals, using two-dimensional diffusion-ordered ^1^H–NMR spectroscopy (2D DOSY). This technique enabled the characterization of hydrodynamic properties specific to each lipoprotein subclass. The diameters of lipoproteins were derived from their diffusion coefficients via the Stokes–Einstein equation [34].

To analyze the methyl signal, nine Lorentzian curves were fitted, each corresponding to a distinct lipoprotein subtype. The area under each Lorentzian peak reflected the lipid content of that subtype, while diffusion data were used to determine particle size. Lipoprotein particle numbers were obtained by dividing the total lipid volume by the estimated volume of a single particle, using established lipid-to-volume conversion factors. The coefficients of variation were between 2% and 4% for particle counts, and below 0.3% for particle sizes ([17] and Supplementary analytical methods).

### 4.3. ^1^H–NMR Data Reporting

Given the complexity of ^1^H–NMR-derived lipoprotein profiles, which involve multiple interrelated parameters, a simplified and intuitive visualization method was required. To address this, we developed the Lipoprotein Score (LS), a novel graphical representation that integrates ten key ^1^H–NMR parameters associated with cardiovascular disease (CVD) risk. In this format, parameters linked to increased atherogenicity cause the LS to shrink inward, with the degree of contraction proportional to the parameter’s deviation from the reference [18]. The magnitude of the shift was determined proportional to the variance of each variable.

In our study, the LS illustrated the mean values of participants within each triglyceride quartile group, depicted by an orange contour, and compared these values with established clinical targets or, when such guidelines were unavailable, with reference intervals derived from the general population (represented by a black circumference). These reference values were obtained from two large Spanish cohorts, the Di@bet.es Study [35] and the Mollerusa Study [36], comprising a total of 6022 individuals. The ten variables incorporated in the LS included: LDL particle diameter (LDL Ø), concentration of small LDL particles (S–LDL–P), LDL cholesterol (LDL–C), LDL triglycerides (LDL–TG), remnant cholesterol (Rem C), VLDL triglycerides (VLDL–TG), HDL particle diameter (HDL Ø), medium-size HDL particle number (M–HDL–P), HDL cholesterol (HDL–C), and the HDL triglyceride-to-cholesterol ratio (HDL–TG/HDL–C).

### 4.4. Statistical Analyses

Patients were divided into quartiles (Q) based on TG levels, ranging from 0.11 to 22.5 mmol/L. The quartile boundaries were defined as follows: Q1: TG < 1.05 mmol/L; Q2: 1.05–1.45 mmol/L; Q3: 1.46–2.17 mmol/L; and Q4: TG > 2.17 mmol/L. To assess the direct impact of sex and the presence of diabetes and obesity (D/O), we conducted separate analyses in women and men and patients with and without D/O. Given the smaller sample size within each subgroup, participants were stratified into tertiles.

All ^1^H–NMR variables were summarized as median and interquartile range (IQR), because their non-normal distribution, which was assessed using the Shapiro–Wilk normality test. Linear trends across quartiles were evaluated using linear regression modeling, with adjustments for sex, age, and body mass index (BMI). Statistical significance was set at α = 0.05. The differences in LS profiles across TG quartiles were visualized using four LS representations, depicting median and IQR profiles for each group.

## Figures and Tables

**Table 1:** Baseline clinical and biochemical characteristics of the studied population sorted by quartiles of triglyceride concentrations (mmol/L).

	All Population					
	All Patients	Quartile 1 <1.05 mmol/L	Quartile 2 1.051–1.45 mmol/L	Quartile 3 1.46–2.17 mmol/L	Quartile 4 >2.17 mmol/L	*p* Value
Number of participants	822	206	205	205	206	
Age (years)	48.3 ± 13.4	44.2 ± 15.1	48.7 ± 12.5	49.6 ± 12.4	50.6 ± 12.7	<0.001
Women (%)	367 (44.8%)	117 (56.8%)	102 (50%)	81 (39.5%)	67 (32.8%)	<0.001
Obesity (%)	259 (31.7%)	29 (14.1%)	50 (24.5%)	68 (33.2%)	112 (54.9%)	<0.001
BMI (kg/m^2^)	27.9 ± 5.14	25.1 ± 4.27	27.3 ± 5	28.3 ± 4.58	30.8 ± 5.01	<0.001
Diabetes (%)	95 (11.6%)	10 (4.85%)	13 (6.34%)	24 (11.7%)	48 (23.3%)	<0.001
Hypertension (%)	164 (20%)	23 (11.2%)	25 (12.2%)	44 (21.5%)	72 (35%)	<0.001
Cardiovascular disease (%)	71 (8.64%)	19 (9.22%)	19 (9.27%)	15 (7.32%)	18 (8.74%)	0.88
Plaque (%)	59 (7.18%)	7 (3.4%)	18 (8.78%)	19 (9.27%)	15 (7.28%)	0.087
	Standard Lipid Profile ^1^					
Total cholesterol (mmol/L)	6.18 (5.17–7.06)	6.03 (4.81–6.88)	6.17 (5.24–6.9)	6.23 (5.43–7.14)	6.36 (5.31–7.31)	0.003
Triglycerides (mmol/L)	1.57 (1.04–2.45)	0.86 (0.69–0.97)	1.28 (1.17–1.45)	1.93 (1.73–2.15)	3.8 (2.83–5.48)	<0.001
LDL–C (mmol/L)	3.92 (2.92–4.68)	3.87 (2.71–4.54)	4.11 (3.2–4.82)	4.03 (3.23–4.86)	3.52 (2.46–4.4)	<0.001
HDL–C (mmol/L)	1.27 (1.03–1.58)	1.55 (1.29–1.93)	1.38 (1.16–1.66)	1.24 (1.06–1.47)	0.98 (0.81–1.16)	<0.001
Apo B–100 (mg/dL)	133 (108–154)	122 (92.8–142)	134 (109–153)	139 (117–162)	137 (114–161)	<0.001
Apo A–I (mg/dL)	144 (127–165)	154 (134–175)	149 (133–166)	143 (129–165)	133 (115–150)	<0.001
Lp(a) (nmol/L)	36.9 (10–138)	37 (11–134)	47 (16–182)	39 (11–138)	23 (7.5–88.5)	<0.001

^1^ Biochemical value: median (IQR).

**Table 2:** Lipoprotein particle number, size, and cholesterol and triglycerides composition determined by ^1^H–NMR spectroscopy (Liposcale) in patients sorted by quartiles of triglyceride serum concentrations ^1^.

	All Population					
	Quartile 1 <1.05 mmol/L	Quartile 2 1.05–1.45 mmol/L	Quartile 3 1.46–2.17 mmol/L	Quartile 4 >2.17 mmol/L	*p*-Trend	*p*-Trend adj.
VLDL–C (mmol/L)	0.12 (0.08–0.19)	0.29 (0.23–0.36)	0.52 (0.44–0.6)	1.12 (0.86–1.62)	<0.001	<0.001
IDL–C (mmol/L)	0.21 (0.16–0.27)	0.3 (0.24–0.35)	0.36 (0.29–0.47)	0.46 (0.34–0.6)	<0.001	<0.001
LDL–C (mmol/L)	3.91 (3.06–4.57)	4.24 (3.49–4.92)	4.25 (3.59–5.19)	3.99 (3.25–4.9)	0.022	0.003
HDL–C (mmol/L)	1.52 (1.28–1.72)	1.39 (1.16–1.57)	1.29 (1.13–1.47)	1.08 (0.94–1.22)	<0.001	<0.001
Remnant C (mmol/L)	0.34 (0.28–0.44)	0.59 (0.51–0.68)	0.89 (0.78–1.02)	1.63 (1.27–2.14)	<0.001	<0.001
Total Cholesterol (mmol/L)	5.89 (4.79–6.58)	6.3 (5.42–7.04)	6.5 (5.68–7.47)	6.82 (5.99–7.99)	<0.001	<0.001
VLDL–TG (mmol/L)	0.45 (0.37–0.52)	0.75 (0.63–0.85)	1.18 (1.02–1.34)	2.54 (1.9–3.85)	<0.001	<0.001
IDL–TG (mmol/L)	0.1 (0.08–0.12)	0.13 (0.11–0.15)	0.16 (0.13–0.19)	0.19 (0.14–0.24)	<0.001	<0.001
LDL–TG (mmol/L)	0.19 (0.14–0.24)	0.23 (0.18–0.27)	0.25 (0.19–0.3)	0.25 (0.18–0.34)	<0.001	<0.001
HDL–TG (mmol/L)	0.12 (0.09–0.15)	0.14 (0.11–0.17)	0.17 (0.13–0.21)	0.2 (0.13–0.26)	<0.001	<0.001
Total Triglycerides (mmol/L)	0.89 (0.78–0.97)	1.24 (1.15–1.33)	1.74 (1.59–1.9)	3.25 (2.53–4.5)	<0.001	<0.001
VLDL–P (nmol/L)	26.2 (22.5–31.9)	45.7 (38.8–52.2)	74.1 (64.8–85.6)	160 (124–234)	<0.001	<0.001
Large VLDL–P (nmol/L)	0.71 (0.56–0.89)	1.09 (0.89–1.31)	1.67 (1.42–1.96)	3.42 (2.68–4.92)	<0.001	<0.001
Medium VLDL–P (nmol/L)	2.79 (2.11–3.38)	4.5 (3.81–5.27)	6.56 (5.17–7.88)	14.9 (9.97–26.7)	<0.001	<0.001
Small VLDL–P (nmol/L)	22.7 (19.3–27.7)	39.8 (33.7–46)	65.7 (56.4–76.9)	138 (111–205)	<0.001	<0.001
LDL–P (nmol/L)	1470 (1179–1688)	1640 (1396–1861)	1700 (1476–2034)	1721 (1452–2100)	<0.001	<0.001
Large LDL–P (nmol/L)	217 (170–256)	235 (193–270)	230 (198–262)	205 (153–248)	0.93	0.14
Medium LDL–P (nmol/L)	521 (335–657)	571 (411–704)	528 (389–689)	394 (252–570)	0.003	0.26
Small LDL–P (nmol/L)	721 (630–807)	832 (743–930)	935 (820–1079)	1104 (889–1280)	<0.001	<0.001
HDL–P (μmol/L)	27.5 (23.6–31.8)	27 (22.9–31)	26 (23.6–29.8)	24.6 (20.8–28.3)	<0.001	0.49
Large HDL–P (μmol/L)	0.28 (0.24–0.32)	0.3 (0.27–0.33)	0.31 (0.27–0.35)	0.29 (0.24–0.34)	0.11	<0.001
Medium HDL–P (μmol/L)	9.76 (8.36–11.4)	9.33 (8.01–10.6)	8.96 (7.86–10.1)	8.2 (6.75–9.61)	<0.001	0.003
Small HDL–P (μmol/L)	17.8 (14.9–20.5)	17.5 (14.6–20)	17.1 (14.6–19.8)	15.8 (13.4–19)	0.052	0.71
VLDL–Z (nm)	42.2 (42–42.3)	42 (41.9–42.2)	41.9 (41.7–42.1)	42 (41.7–42.3)	0.012	0.019
LDL–Z (nm)	21.2 (21–21.4)	21.1 (20.9–21.4)	20.9 (20.8–21.2)	20.6 (20.3–20.8)	<0.001	<0.001
HDL–Z (nm)	8.28 (8.23–8.33)	8.26 (8.22–8.31)	8.26 (8.21–8.31)	8.25 (8.19–8.31)	0.19	0.3
HDL–TG/HDL–C	0.18 (0.14–0.22)	0.23 (0.19–0.28)	0.29 (0.23–0.36)	0.41 (0.29–0.59)	<0.001	<0.001

^1^ Data are reported as median (interquartile range). The p-trend adjusted values presented are adjusted to control for the confounding effects of age, sex, and body mass index, based on regression models for each variable reported. Additional abbreviations: –C: cholesterol concentration; –TG: triglyceride concentrations; –P: particle number; –Z: particle size (diameter). mmol/L: millimole/liter; nmol/L: nanomole/liter; mmol/L: micromole/liter. Biochemical values: median (IQR).

**Table 3:** Baseline clinical and biochemical characteristics of the studied population categorized by the absence or presence of diabetes and obesity (D/O) and further stratified into tertiles according to serum triglyceride concentrations (mmol/L).

	No DM and BMI < 30 (*n* = 527)				DM or BMI > 30 (*n* = 287)			
Variable	Tertile 1 <1.1 mmol/L	Tertile 2 1.1–1.6 mmol/L	Tertile 3 >1.6 mmol/L	*p* Value	Tertile 1 <1.5 mmol/L	Tertile 2 1.5–2.5 mmol/L	Tertile 3 >2.5 mmol/L	*p* Value
Number of participants	176	175	176		96	95	96	
Age (years)	42.9 ± 14.5	46.8 ± 12.8	48.6 ± 12.5	<0.001	50.9 ± 14.2	52.4 ± 11	52.3 ± 12.6	0.81
Women (%)	104 (59.1%)	86 (49.1%)	66 (37.5%)	<0.001	49 (51%)	32 (33.7%)	28 (29.2%)	0.004
BMI (kg/m^2^)	23.9 ± 3.1	25 ± 2.8	26.3 ± 2.41	<0.001	32.1 ± 4.8	32.8 ± 3.82	34.1 ± 4.17	0.004
Hypertension (%)	11 (6.25%)	15 (8.57%)	27 (15.3%)	0.013	24 (25%)	41 (43.2%)	46 (47.9%)	0.003
Cardiovascular disease (%)	14 (7.95%)	13 (7.43%)	7 (3.98%)	0.26	12 (12.5%)	12 (12.6%)	11 (11.5%)	0.96
Plaque (%)	6 (3.41%)	16 (9.14%)	12 (6.82%)	0.089	7 (7.29%)	7 (7.37%)	9 (9.38%)	0.83
	Standard Lipid Profile ^1^							
Total cholesterol (mmol/L)	6.1 (4.81–6.91)	6.36 (5.59–6.98)	6.44 (5.6–7.29)	0.002	5.53 (4.72–6.59)	5.87 (4.69–6.93)	6.54 (5.42–7.68)	<0.001
Triglycerides (mmol/L)	0.85 (0.68–0.95)	1.35 (1.21–1.52)	2.37 (2.01–3.07)	<0.001	1.2 (0.99–1.38)	2.03 (1.82–2.39)	4.74 (3.56–6.35)	<0.001
LDL–C (mmol/L)	3.9 (2.77–4.56)	4.22 (3.47–4.93)	4.03 (3.13–4.87)	0.012	3.7 (2.77–4.48)	3.85 (2.66–4.5)	3.54 (2.54–4.16)	0.12
HDL–C (mmol/L)	1.59 (1.37–2)	1.42 (1.22–1.71)	1.14 (0.96–1.34)	<0.001	1.32 (1.11–1.58)	1.16 (0.93–1.36)	0.93 (0.76–1.1)	<0.001
Apo B–100 (mg/dL)	122 (91–141)	137 (116–157)	139 (119–165)	<0.001	122 (98–143)	138 (106–154)	140 (118–160)	<0.001
Apo A–I (mg/dL)	156 (140–180)	152 (134–172)	139 (126–160)	<0.001	141 (131–160)	140 (120–162)	130 (113–145)	0.001
Lp(a) (nmol/L)	45 (16.9–155)	47 (14.2–170)	21 (8.45–89.2)	<0.001	34 (10.1–127)	44 (13.5–142)	27 (9–81)	0.25

^1^ Biochemical values: median (IQR).

**Table 4:** Lipoprotein particle number, size, and cholesterol and triglycerides composition determined by ^1^H–NMR spectroscopy (Liposcale) in patients categorized by the absence or presence of diabetes and obesity (D/O) and further stratified into tertiles according to serum triglyceride concentrations (mmol/L) ^1^.

	No DM and BMI < 30 (*n* = 527)					DM or BMI > 30 (*n* = 287)				
Variable	Tertile 1	Tertile 2	Tertile 3	*p*-Trend	*p*-Trend adj.	Tertile 1	Tertile 2	Tertile 3	*p*-Trend	*p*-Trend adj.
VLDL–C (mmol/L)	0.11 (0.08–0.18)	0.32 (0.25–0.39)	0.7 (0.54–1.05)	<0.001	<0.001	0.28 (0.18–0.35)	0.53 (0.46–0.72)	1.3 (1.01–1.76)	<0.001	<0.001
IDL–C (mmol/L)	0.21 (0.16–0.26)	0.31 (0.24–0.39)	0.44 (0.32–0.55)	<0.001	<0.001	0.26 (0.2–0.32)	0.34 (0.28–0.44)	0.48 (0.35–0.68)	<0.001	<0.001
LDL–C (mmol/L)	3.99 (3.04–4.59)	4.3 (3.81–5.01)	4.26 (3.57–5.16)	0.002	<0.001	3.75 (3.28–4.54)	4.04 (3.39–4.85)	3.98 (3.38–4.94)	0.048	0.035
HDL–C (mmol/L)	1.54 (1.31–1.75)	1.43 (1.2–1.61)	1.19 (1.07–1.39)	<0.001	<0.001	1.34 (1.1–1.55)	1.22 (1.08–1.37)	1.05 (0.85–1.18)	0.001	0.01
Remnant C (mmol/L)	0.33 (0.27–0.44)	0.62 (0.55–0.74)	1.14 (0.93–1.47)	<0.001	<0.001	0.52 (0.4–0.67)	0.92 (0.82–1.04)	1.87 (1.55–2.46)	<0.001	<0.001
Cholesterol (mmol/L)	6.09 (4.79–6.61)	6.43 (5.81–7.26)	6.84 (5.91–7.76)	<0.001	<0.001	5.61 (4.98–6.62)	6.14 (5.46–7.14)	6.91 (6.21–8.24)	<0.001	<0.001
VLDL–TG (mmol/L)	0.43 (0.36–0.51)	0.77 (0.64–0.87)	1.48 (1.23–2.1)	<0.001	<0.001	0.66 (0.55–0.82)	1.28 (1.12–1.64)	3.35 (2.43–4.64)	<0.001	<0.001
IDL–TG (mmol/L)	0.1 (0.09–0.11)	0.13 (0.11–0.16)	0.18 (0.14–0.21)	<0.001	<0.001	0.12 (0.1–0.13)	0.16 (0.13–0.19)	0.19 (0.15–0.24)	<0.001	<0.001
LDL–TG (mmol/L)	0.2 (0.14–0.24)	0.24 (0.2–0.29)	0.27 (0.19–0.34)	<0.001	<0.001	0.19 (0.16–0.25)	0.23 (0.19–0.27)	0.25 (0.16–0.32)	<0.001	<0.001
HDL–TG (mmol/L)	0.12 (0.1–0.15)	0.15 (0.12–0.18)	0.18 (0.14–0.24)	<0.001	<0.001	0.13 (0.1–0.16)	0.17 (0.13–0.2)	0.18 (0.08–0.26)	<0.001	<0.001
Triglycerides (mmol/L)	0.88 (0.78–0.97)	1.29 (1.17–1.43)	2.16 (1.82–2.72)	<0.001	<0.001	1.14 (0.96–1.33)	1.84 (1.66–2.13)	3.99 (3.14–5.33)	<0.001	<0.001
VLDL–P (nmol/L)	25.5 (22.2–31.3)	47.3 (39.1–55.6)	96.4 (78.8–138)	<0.001	<0.001	41.7 (33.3–51.6)	82 (69.7–101)	205 (152–289)	<0.001	<0.001
Large VLDL–P (nmol/L)	0.68 (0.56–0.83)	1.08 (0.91–1.31)	2.15 (1.75–2.97)	<0.001	<0.001	1.1 (0.84–1.32)	1.91 (1.58–2.35)	4.32 (3.29–6.2)	<0.001	<0.001
Medium VLDL–P (nmol/L)	2.8 (2.06–3.4)	4.56 (3.85–5.41)	8.4 (6.37–12.6)	<0.001	<0.001	4.13 (3.1–5.15)	7.02 (5.71–8.67)	21.8 (14.1–33.1)	<0.001	<0.001
Small VLDL–P (nmol/L)	21.9 (19–27)	41.3 (34.1–49.3)	85.7 (70.4–123)	<0.001	<0.001	36.7 (28.9–45.4)	71.8 (61.4–90.8)	175 (136–249)	<0.001	<0.001
LDL–P (nmol/L)	1482 (1168–1702)	1684 (1482–1894)	1746 (1513–2067)	<0.001	<0.001	1488 (1282–1708)	1652 (1418–1990)	1739 (1479–2178)	<0.001	<0.001
Large LDL–P (nmol/L)	228 (170–261)	238 (205–272)	230 (190–264)	0.15	0.01	208 (170–245)	213 (169–244)	197 (150–245)	0.61	0.66
Medium LDL–P (nmol/L)	533 (330–672)	589 (460–711)	489 (354–674)	0.87	0.32	473 (382–635)	473 (335–630)	360 (240–534)	0.06	0.12
Small LDL–P (nmol/L)	719 (627–803)	842 (741–957)	996 (879–1177)	<0.001	<0.001	785 (686–897)	959 (828–1083)	1148 (892–1346)	<0.001	<0.001
HDL–P (μmol/L)	28.4 (24.6–32.2)	27.7 (23.8–31.3)	25.5 (23.1–29.5)	0.007	0.21	25.7 (21.9–29.3)	25.2 (22.8–27.6)	22.8 (18.9–27)	0.19	0.6
Large HDL–P (μmol/L)	0.29 (0.25–0.33)	0.31 (0.27–0.34)	0.31 (0.27–0.35)	0.002	<0.001	0.28 (0.25–0.31)	0.29 (0.26–0.32)	0.27 (0.21–0.33)	0.7	0.89
Medium HDL–P (μmol/L)	10 (8.52–11.6)	9.58 (8.18–10.9)	8.87 (7.56–10.2)	<0.001	0.0063	9.15 (7.81–10.3)	8.47 (7.48–9.49)	7.86 (6.19–9.04)	0.009	0.048
Small HDL–P (μmol/L)	18.2 (15.5–20.8)	17.9 (15.2–21.2)	16.7 (14.4–19.5)	0.23	0.66	16.4 (13.6–19.3)	16.9 (14.2–18.7)	15.6 (11.9–18.3)	0.57	0.87
VLDL–Z (nm)	42.2 (42–42.3)	42 (41.9–42.2)	41.9 (41.7–42.2)	<0.001	<0.001	42 (41.9–42.2)	41.9 (41.7–42.1)	42.1 (41.9–42.4)	0.12	0.23
LDL–Z (nm)	21.2 (21–21.5)	21.2 (20.9–21.4)	20.8 (20.6–21.1)	<0.001	<0.001	21.1 (20.9–21.3)	20.8 (20.6–21.1)	20.4 (20.1–20.8)	<0.001	<0.001
HDL–Z (nm)	8.27 (8.23–8.32)	8.27 (8.22–8.31)	8.25 (8.21–8.3)	0.048	0.66	8.28 (8.23–8.33)	8.26 (8.21–8.3)	8.24 (8.18–8.31)	0.4	0.24
HDL–TG/HDL–C	0.18 (0.14–0.22)	0.24 (0.19–0.29)	0.35 (0.26–0.47)	<0.001	<0.001	0.22 (0.17–0.29)	0.32 (0.23–0.39)	0.37 (0.21–0.62)	<0.001	<0.001

^1^ Data are reported as median (interquartile range). The *p*-trend adjusted values presented are adjusted to control for the confounding effects of age, sex, and body mass index, based on regression models for each variable reported. Additional abbreviations: –C: cholesterol concentration; –TG: triglyceride concentrations; –P: particle number; –Z: particle size (diameter). mmol/L: millimole/liter; nmol/L: nanomole/liter; mmol/L: micromole/liter.

## Data Availability

The data underlying this article will be shared on reasonable request to the corresponding author.

## Supplementary Materials

The following supporting information can be downloaded at: https://www.mdpi.com/article/10.3390/ijms26178284/s1.

## Abbreviations

The following abbreviations are used in this manuscript:

apoB	Apolipoprotein B
ASCVD	Atherosclerotic Cardiovascular Disease
CETP	Cholesteryl Ester Transfer Protein
CV	Cardiovascular
D/O	Diabetes and/or Obesity
FA	Fatty Acid
FCS	Familial Chylomicronemia Syndrome
HDL	High-Density Lipoprotein
HDL–C	HDL Cholesterol
HDL–TG	HDL Triglycerides
HDL Ø	HDL particle diameter
M–HDL–P	Medium-size HDL particle number
HDL–TG/HDL–Ch	HDL Triglyceride-to-Cholesterol Ratio
IDL	Intermediate-Density Lipoprotein
IQR	Interquartile Range
LED	Longitudinal Eddy Current Delay
LLT	Lipid-Lowering Therapy
Lp(a)	Lipoprotein(a)
LS	Lipid Silhouette
LDL	Low-Density Lipoprotein
LDL–C	LDL Cholesterol
LDL–TG	LDL Triglycerides
LDL Ø	LDL particle diameter
M–HDL–P	Medium HDL Particles
NMR	Nuclear Magnetic Resonance
NOESY	Nuclear Overhauser Effect Spectroscopy
PPAR–a/g	peroxisome proliferator–activated receptor–α/γ agonist
Q1–Q4	Quartiles 1 through 4
Rm–Chol	Remnant Cholesterol
S–LDL–P	Smaller LDL Particles
TG	Triglycerides
TRL	Triglyceride-Rich Lipoprotein
VLDL	Very Low-Density Lipoprotein
VLDL–TG	VLDL Triglycerides
VLDL Ø	VLDL particle diameter
