# Supplementary material for: Triglycerides as Determinants of Global Lipoprotein Derangement: Implications for Cardiovascular Prevention
Source: Int J Mol Sci. 2025 Aug 26;26(17):8284. doi: 10.3390/ijms26178284 (PMC12427746; doi:10.3390/ijms26178284)
Supplement: Supplementary file 1 [file ijms-26-08284-s001.zip › ijms-3814373-supplementary.pdf]

SUPPLEMENTARY ^{1}H-NMR ANALYTICAL METHODS

Quantitative ^{1}H-NMR Lipoprotein Profiling .

Liposcale method (IVD-CE marked), was used to determine the particle size and number of nine lipoprotein subtypes: large, medium, and small very low-density lipoprotein (VLDL), low-density lipoprotein (LDL), and high-density lipoprotein (HDL). Cholesterol and TG concentrations in lipoprotein subclasses were also measured. Remnant cholesterol (Rm-Chol) was calculated as the sum of IDL and VLDL cholesterol.

An internal control sample derived from a pooled human serum was used to ensure the **quantitative** nature and quality assurance throughout the year. Human serum aliquots, each 200 µL, were prepared and stored at -80ºC. Prior to preparation, the samples were completely thawed (minimum of 1 hour), and the serum was homogenized by inverting the tubes twice. Diluted samples were then prepared by mixing **200 µL of serum, 300 µL of 50 mM phosphate buffer (pH 7.4), and 50 µL of deuterated water** before being transferred to 5 mm 1H-NMR tubes. The tubes were carefully maintained below 8ºC until they were introduced into the 1H-NMR spectrometer.
Before any measurement, we allowed the sample to equilibrate for at least 5 minutes after its introduction into the spectrometer to ensure temperature stability. Once stabilized, the acquisition was fully automated, including equilibration, tuning, matching, pulse calibration, fixed receiver gain, and number of scans. For sample referencing, digital ERETIC (Electronic Reference To access In Vivo Concentrations, Bruker®) was pre-calibrated against a reference sample and adjusted during the NMR measurement, accounting for variations in the 90º pulse length, number of scans (NS), and receiver gain (RG), following automatic tuning and matching.
To ensure consistent performance of the Liposcale® test, a control aliquot was analyzed at the start of each day. The variability in the spectra should be <1% within the region between 1.9 and 0.4 ppm. As illustrated in Figure below, NMR spectra from different days were overlapped using the Multiple Display function in TopSpin 3.2 (Bruker) to verify that no intensity changes were found.
Automatic processing of the spectrum, integrated within the Liposcale® software, included three steps: phase correction, baseline correction, and referencing. The spectrum was referenced and aligned within the lipoprotein region This processing ensured that all spectra were analyzed under consistent conditions.
a) Reference alignment:

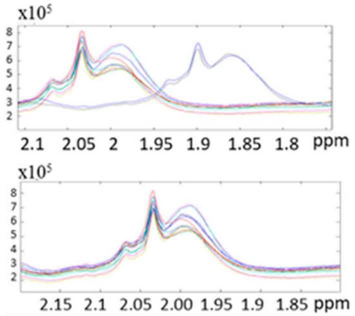

b) Baseline correction:

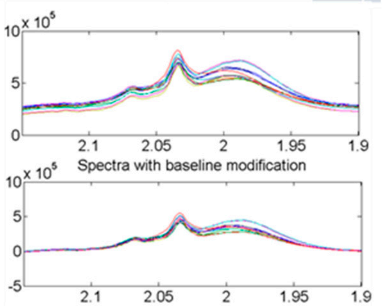

The deconvolution of the methyl group signals, which appear around 0.8 ppm, is performed on the processed spectrum. Each deconvolution function is associated with a specific lipoprotein type (VLDL, LDL, or HDL) and a corresponding subparticle size. The primary lipoprotein fractions are defined within the following size ranges: 38.6-81.9 nm for VLDL, 14.7-26.6 nm for LDL, and 6.0-10.9 nm for HDL. The configuration of the resulting deconvolution functions provides information about the proportion of each lipoprotein subtype present in the sample. The average particle size for each main lipoprotein type (VLDL, LDL, and HDL) is determined by averaging the area under each function according to its associated size.

SUPPLEMENTARY TABLE.

Main Clinical variables, standard lipid profile and lipoprotein profile assessed by 1H–NMR in women and men sorted by triglyceride tertiles.

Variable	Women					Men				
	All Patients	Tertile 1 < 1.07 mmol/L	Tertile 2 1.07–1.58 mmol/L	Tertile 3 >1.58 mmol/L	p value	All Patients	Tertile 1 < 1.31 mmol/L	Tertile 2 1.31–2.06 mmol/L	Tertile 3 >2.06 mmol/L	p value
Number of participants	367	123	122	122		455	152	151	152	
Age (years)	49.2 ± 14.1	45.4 ± 14.1	49.8 ± 13.9	52.5 ± 13.6	<0.001	47.4 ± 12.8	44.5 ± 14	47.6 ± 11.9	50.1 ± 11.9	0.002
Obesity (%)	98 (26.7%)	15 (12.2%)	33 (27%)	50 (41%)	<0.001	162 (35.6%)	25 (16.4%)	50 (33.1%)	87 (57.2%)	<0.001
BMI (kg/m2)	26.9 ± 5.47	24.7 ± 4.34	26.9 ± 5.68	29.3 ± 5.33	<0.001	28.6 ± 4.74	26.3 ± 4.08	28.6 ± 4.46	31 ± 4.51	<0.001
Diabetes (%)	30 (8.17%)	3 (2.44%)	10 (8.2%)	17 (13.9%)	0.005	65 (14.3%)	10 (6.58%)	16 (10.6%)	39 (25.7%)	<0.001
Hypertension (%)	55 (15%)	10 (8.13%)	13 (10.7%)	32 (26.2%)	<0.001	110 (24.2%)	22 (14.5%)	36 (23.8%)	52 (34.2%)	<0.001
Cardiovascular disease (%)	18 (4.9%)	5 (4.07%)	8 (6.56%)	5 (4.1%)	0.59	53 (11.6%)	24 (15.8%)	12 (7.95%)	17 (11.2%)	0.1
Plaque (%)	27 (7.36%)	4 (3.25%)	10 (8.2%)	13 (10.7%)	0.077	31 (6.81%)	10 (6.58%)	12 (7.95%)	9 (5.92%)	0.78
	Standard Lipid Profile									
Total cholesterol (mmol/L)	6.49 (5.51–7.32)	6.34 (5.3–7.03)	6.54 (5.54–7.39)	6.8 (5.82–7.73)	0.008	5.97 (4.94–6.84)	5.68 (4.38–6.49)	6.03 (5–6.9)	6.19 (5.14–7.07)	0.001
Triglycerides (mmol/L)	1.31 (0.95–2.02)	0.86 (0.69–0.97)	1.32 (1.16–1.48)	2.53 (2.02–3.44)	<0.001	1.8 (1.21–2.78)	1.02 (0.82–1.22)	1.82 (1.57–2.04)	3.71 (2.76–5.51)	<0.001
LDL–C (mmol/L)	4.11 (3.34–4.87)	4.06 (3.28–4.63)	4.14 (3.39–5.18)	4.14 (3.3–4.94)	0.41	3.78 (2.66–4.5)	3.74 (2.28–4.48)	3.9 (3–4.68)	3.47 (2.35–4.14)	0.004
HDL–C (mmol/L)	1.5 (1.22–1.81)	1.68 (1.44–2.16)	1.58 (1.34–1.81)	1.23 (1.05–1.5)	<0.001	1.14 (0.93–1.37)	1.37 (1.16–1.58)	1.14 (1.03–1.34)	0.91 (0.78–1.11)	<0.001
Apo B–100 (mg/dL)	136 (114–158)	126 (107–146)	135 (109–160)	146 (128–170)	<0.001	132 (105–149)	122 (89.2–142)	135 (113–154)	132 (110–153)	0.001
Apo A–I (mg/dL)	159 (139–185)	162 (138–190)	163 (146–186)	152 (134–175)	0.093	136 (118–151)	144 (130–158)	136 (125–151)	126 (112–144)	<0.001
Lp(a) (nmol/L)	37.5 (11.1–138)	38.5 (12.6–116)	48.5 (18.8–193)	26 (8.35–104)	0.006	36.2 (9.55–134)	44 (12.7–170)	38 (11.4–138)	21 (7.92–78.3)	0.002

Variable	Women (n=367) Tertile 1 < 1.07 mmol/L	Women Tertile 2 1.07–1.58 mmol/L	Women Tertile 3 >1.58 mmol/L	p–trend	p–trend adj.	Men (n=455) Tertile 1 < 1.31 mmol/L	Men Tertile 2 1.31–2.06 mmol/L	Men Tertile 3 >2.06 mmol/L	p–trend	p–trend adj.
VLDL–C (mmol/L)	0.1 (0.08–0.17)	0.29 (0.21–0.36)	0.74 (0.52–1.15)	<0.001	<0.001	0.2 (0.13–0.28)	0.49 (0.41–0.56)	1.05 (0.83–1.62)	<0.001	<0.001
IDL–C (mmol/L)	0.22 (0.17–0.27)	0.33 (0.27–0.41)	0.48 (0.39–0.57)	<0.001	<0.001	0.23 (0.17–0.29)	0.32 (0.24–0.4)	0.42 (0.32–0.57)	<0.001	<0.001
LDL–C (mmol/L)	4.2 (3.39–4.68)	4.35 (3.7–5.14)	4.49 (3.64–5.29)	0.002	0.013	3.77 (2.87–4.48)	4.13 (3.54–4.85)	3.85 (3.22–4.68)	0.16	0.077
HDL–C (mmol/L)	1.64 (1.44–1.9)	1.52 (1.34–1.77)	1.28 (1.11–1.5)	<0.001	<0.001	1.33 (1.14–1.5)	1.21 (1.08–1.36)	1.06 (0.89–1.22)	<0.001	0.003
Remnant C (mmol/L)	0.34 (0.28–0.43)	0.6 (0.54–0.74)	1.21 (0.98–1.72)	<0.001	<0.001	0.44 (0.32–0.56)	0.81 (0.69–0.94)	1.6 (1.19–2.12)	<0.001	<0.001
Total Cholesterol (mmol/L)	6.23 (5.33–6.75)	6.61 (5.95–7.44)	7.11 (6.39–8.04)	<0.001	<0.001	5.6 (4.6–6.32)	6.18 (5.41–7.09)	6.64 (5.79–7.51)	<0.001	<0.001
VLDL–TG (mmol/L)	0.42 (0.35–0.5)	0.69 (0.61–0.84)	1.53 (1.16–2.25)	<0.001	<0.001	0.57 (0.46–0.72)	1.12 (0.98–1.25)	2.49 (1.87–3.9)	<0.001	<0.001
IDL–TG (mmol/L)	0.1 (0.09–0.12)	0.14 (0.12–0.16)	0.19 (0.16–0.22)	<0.001	<0.001	0.11 (0.09–0.12)	0.14 (0.12–0.17)	0.17 (0.13–0.21)	<0.001	<0.001
LDL–TG (mmol/L)	0.21 (0.15–0.25)	0.25 (0.21–0.3)	0.28 (0.23–0.34)	<0.001	<0.001	0.18 (0.14–0.23)	0.23 (0.18–0.27)	0.24 (0.16–0.29)	<0.001	<0.001
HDL–TG (mmol/L)	0.12 (0.1–0.16)	0.16 (0.14–0.19)	0.21 (0.16–0.26)	<0.001	<0.001	0.12 (0.09–0.14)	0.14 (0.12–0.18)	0.18 (0.12–0.24)	<0.001	<0.001
Total Triglycerides (mmol/L)	0.89 (0.78–0.97)	1.27 (1.17–1.42)	2.26 (1.86–2.97)	<0.001	<0.001	1 (0.87–1.14)	1.65 (1.45–1.79)	3.14 (2.42–4.5)	<0.001	<0.001
VLDL–P (nmol/L)	24.8 (21.3–30.2)	43.8 (37.9–52.5)	96.1 (77.4–147)	<0.001	<0.001	35.3 (27.3–44.9)	70.7 (59.4–79.7)	151 (118–241)	<0.001	<0.001
Large VLDL–P (nmol/L)	0.62 (0.54–0.79)	1.01 (0.82–1.27)	2.18 (1.62–3.18)	<0.001	<0.001	0.96 (0.74–1.16)	1.65 (1.39–1.87)	3.42 (2.6–5.05)	<0.001	<0.001
Medium VLDL–P (nmol/L)	2.79 (2.11–3.36)	4.47 (3.71–5.23)	8.7 (6.38–14.2)	<0.001	<0.001	3.4 (2.44–4.34)	6.35 (4.75–7.72)	14.8 (9.44–26.8)	<0.001	<0.001
Small VLDL–P (nmol/L)	21.2 (18.1–26.5)	37.7 (32.6–46)	85.7 (66.4–130)	<0.001	<0.001	31.2 (24–39.1)	62.5 (52.5–70.9)	135 (106–209)	<0.001	<0.001
LDL–P (nmol/L)	1562 (1287–1716)	1648 (1460–1947)	1840 (1546–2176)	<0.001	<0.001	1460 (1160–1717)	1640 (1454–1878)	1662 (1420–1990)	<0.001	<0.001
Large LDL–P (nmol/L)	234 (188–264)	248 (210–286)	234 (194–272)	0.36	0.21	205 (161–246)	220 (192–248)	200 (150–236)	0.69	0.3
Medium LDL–P (nmol/L)	574 (416–695)	629 (483–754)	545 (375–714)	0.98	0.78	452 (307–609)	498 (368–624)	364 (248–505)	0.033	0.14
Small LDL–P (nmol/L)	721 (644–800)	797 (710–905)	1015 (898–1214)	<0.001	<0.001	774 (647–917)	912 (827–1055)	1039 (845–1274)	<0.001	<0.001
HDL–P (µmol/L)	29.5 (26.7–33.9)	29.3 (26–34.3)	27.3 (23.9–31.9)	0.017	0.14	25.3 (22.3–28.3)	24.4 (22.2–27.2)	24 (20–26.7)	0.25	0.98
Large HDL–P (µmol/L)	0.3 (0.26–0.34)	0.32 (0.29–0.37)	0.31 (0.28–0.36)	0.033	0.006	0.27 (0.23–0.3)	0.29 (0.26–0.33)	0.28 (0.23–0.33)	0.1	0.058
Medium HDL–P (µmol/L)	10.7 (9.54–12.2)	10.2 (9.11–11.5)	9.48 (8.06–10.5)	<0.001	<0.001	8.53 (7.69–9.8)	8.23 (7.3–9.41)	7.98 (6.66–9.18)	0.025	0.19
Small HDL–P (µmol/L)	18.7 (15.9–21.7)	18.9 (16.5–22.5)	18.2 (15.7–20.4)	0.45	0.8	16.3 (13.9–18.7)	15.7 (14–18.2)	15.5 (12.8–18.4)	0.61	0.6
VLDL–Z (nm)	42.2 (42–42.4)	42 (41.9–42.2)	41.9 (41.7–42.1)	<0.001	<0.001	42.1 (41.9–42.3)	41.9 (41.8–42.1)	42 (41.8–42.3)	0.27	0.4
LDL–Z (nm)	21.3 (21.1–21.5)	21.3 (21.1–21.5)	20.9 (20.5–21.1)	<0.001	<0.001	21.1 (20.8–21.2)	20.9 (20.7–21.1)	20.6 (20.3–20.8)	<0.001	<0.001

HDL–Z (nm)	8.29 (8.25–8.33)	8.26 (8.23–8.31)	8.25 (8.2–8.31)	0.025	0.14	8.26 (8.22–8.31)	8.27 (8.21–8.32)	8.25 (8.19–8.31)	0.71	0.79
HDL–TG/HDL–C	0.18 (0.14–0.21)	0.25 (0.2–0.29)	0.37 (0.28–0.5)	<0.001	<0.001	0.2 (0.15–0.24)	0.27 (0.21–0.33)	0.38 (0.24–0.52)	<0.001	<0.001
