# Supplementary figures and images for: Triglycerides as Determinants of Global Lipoprotein Derangement: Implications for Cardiovascular Prevention
Source: Int J Mol Sci. 2025 Aug 26;26(17):8284. doi: 10.3390/ijms26178284 (PMC12427746; doi:10.3390/ijms26178284)

Supplementary figure S1

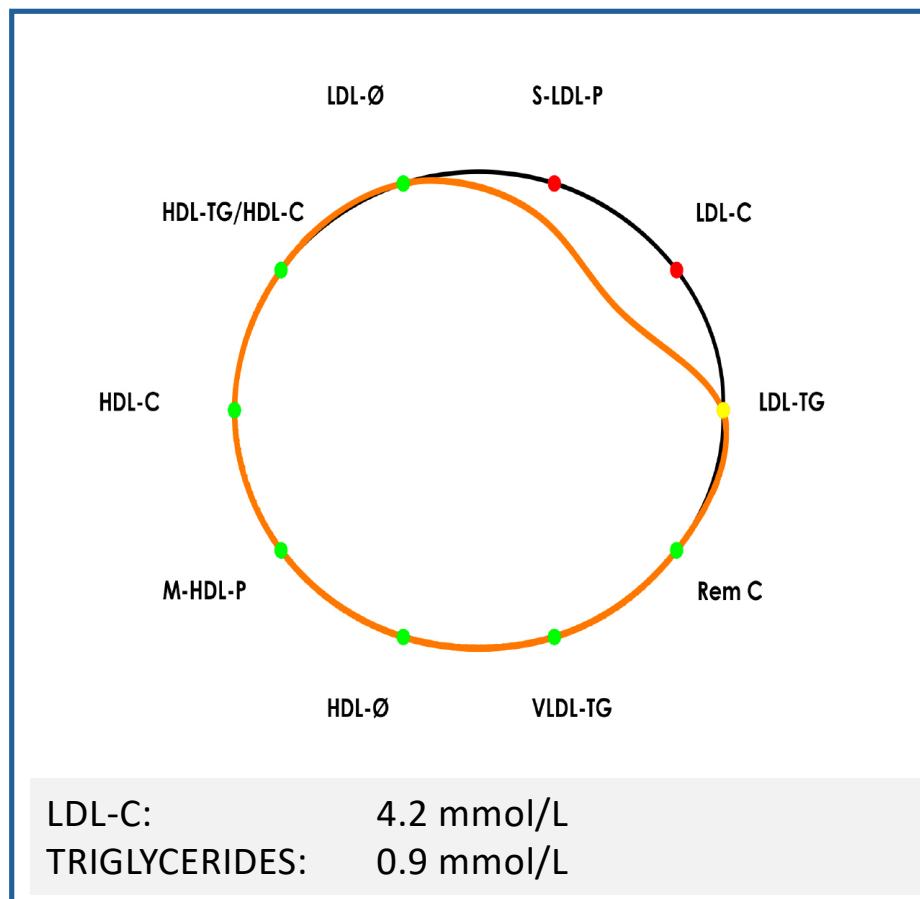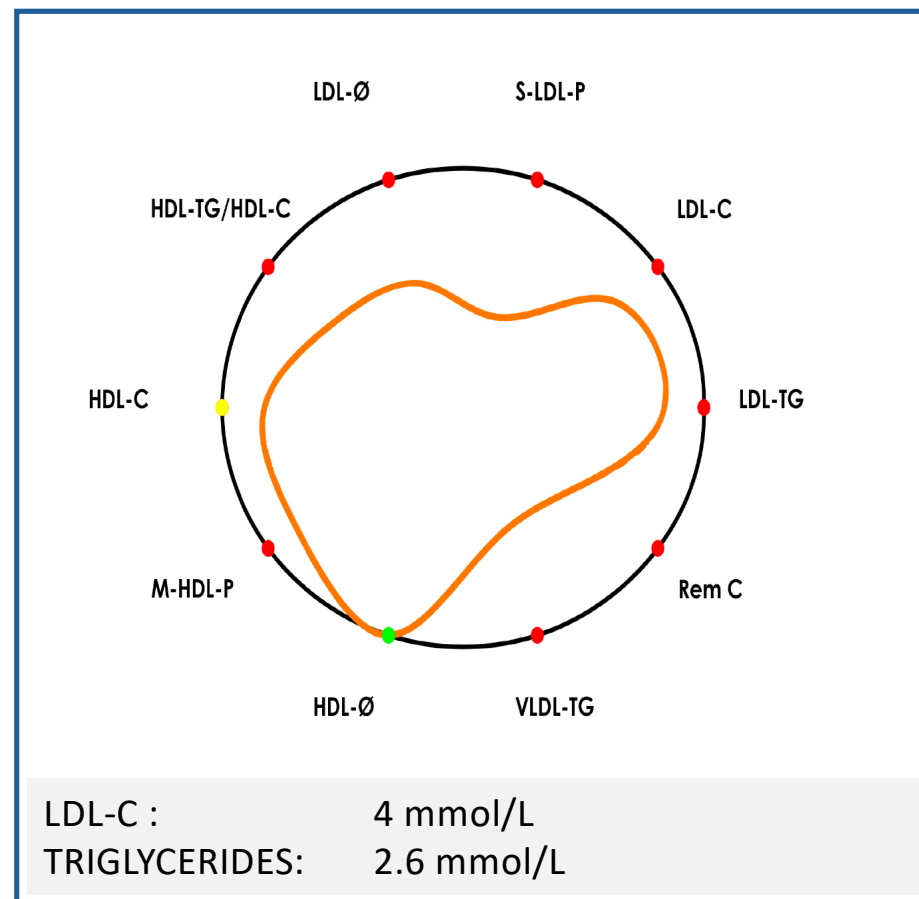

Supplement: Supplementary file 1 [file ijms-26-08284-s001.zip › ijms-3814373-supplementary figure.pdf]
